# Between *μ*-*b*-open sets and *μ*-*β*-open sets in generalized topological spaces

**DOI:** 10.1016/j.heliyon.2023.e13596

**Published:** 2023-02-09

**Authors:** Mohammad S. Sarsak

**Affiliations:** Department of Mathematics, Faculty of Science, The Hashemite University, P.O. Box 330127, Zarqa 13133, Jordan

**Keywords:** primary, 54A05, 54A10, Generalized topology, *μ*-*b*-open, *μ*-*β*-open, $\bar{\mu }$-open

## Abstract

We introduce and study a new class of generalized open sets in generalized topological spaces, called $\bar{\mu }$-open sets. $\bar{\mu }$-open sets are strictly placed between *μ*-*b*-open sets and *μ*-*β*-open sets. Several properties and characterizations of $\bar{\mu }$-open sets are studied extensively.

## 1 Introduction

Let *X* be a non-empty set with power set exp⁡X. A collection μ⊂exp⁡X is called a generalized topology (GT) [1] on *X* if ∅∈μ and each union of a non-empty subclass of *μ* belongs to *μ*. For a GT *μ* on *X*, the pair (X,μ) will be called a generalized topological space (GTS), elements of *μ* will be called *μ*-open sets, and a subset *A* of (X,μ) will be called *μ*-closed if X﹨A is *μ*-open. A space (X,μ) will always mean a GTS. If *A* is a subset of a space (X,μ), then the *μ*-closure of *A* [2], $c_{\mu }\left(A\right)$, is the intersection of all *μ*-closed sets containing *A*, and the *μ*-interior of *A* [2], $i_{\mu }\left(A\right)$, is the union of all *μ*-open sets contained in *A*.

A GT *μ* on *X* is called a strong GT [3] if X∈μ; a space (X,μ) will be called a strong GTS whenever *μ* is a strong GT. Strong GTs were introduced by Lugojan [4] (resp. Mashhour et al. [5], Noiri and Popa [6]) under the name generalized topologies (resp. supra-topologies, minimal structures); Noiri [7] called a space (X,μ), with X∈μ, a *μ*-space. The term “minimal structure” was used sometimes in literature to mean a collection *μ* in which the only assumption is that ∅,X∈μ (see e.g. [8] and [9]).

In GTSs, several types of generalized open sets have been studied; the primary purpose of this article is to introduce and study a new type of generalized open sets in GTSs, called $\bar{\mu }$-open sets. In Section 3, we study several properties and characterizations of $\bar{\mu }$-open sets as well as $\bar{\mu }$-closed sets; the collection $\bar{\mu }$ consisting of all $\bar{\mu }$-open sets forms a strong GT; $\bar{\mu }$-open sets are strictly placed between *μ*-*b*-open sets and *μ*-*β*-open sets. In Section 4, we obtain several decompositions of *μ*-regular open sets and regular *μ*-semi-open sets in terms of $\bar{\mu }$-open sets; we also provide several characterizations of spaces in which every $\bar{\mu }$-open set is *μ*-preopen.

## 2 Preliminaries

A subset *A* of a space (X,μ) is called *μ*-semi-open [2] (resp. *μ*-preopen [2], *μ*-*β*-open [2], *μ*-*α*-open [2], *μ*-*b*-open [10], *μ*-regular closed [11]) if $A\subset c_{\mu }\left(i_{\mu }\left(A\right)\right)$ (resp. $A\subset i_{\mu }\left(c_{\mu }\left(A\right)\right)$, $A\subset c_{\mu }\left(i_{\mu }\left(c_{\mu }\left(A\right)\right)\right)$, $A\subset i_{\mu }\left(c_{\mu }\left(i_{\mu }\left(A\right)\right)\right)$, $A\subset i_{\mu }\left(c_{\mu }\left(A\right)\right)\cup c_{\mu }\left(i_{\mu }\left(A\right)\right)$, $A=c_{\mu }\left(i_{\mu }\left(A\right)\right)$); *A* is called [11] *μ*-semi-closed (resp. *μ*-preclosed, *μ*-*β*-closed, *μ*-*α*-closed, *μ*-regular open) if X﹨A is *μ*-semi-open (resp. *μ*-preopen, *μ*-*β*-open, *μ*-*α*-open, *μ*-regular closed); and *A* is called [10] *μ*-*b*-closed if X﹨A is *μ*-*b*-open and called regular *μ*-semi-open if there exists a *μ*-regular open set *U* such that $U\subset A\subset c_{\mu }\left(U\right)$.

For a space (X,μ), we will denote the class of *μ*-semi-open sets by *σ*, the class of *μ*-preopen sets by *π*, the class of *μ*-*β*-open sets by *β*, the class of *μ*-*α*-open sets by *α*, and the class of *μ*-*b*-open sets by $b_{\mu }$ or *b*; we will also denote the class of regular *μ*-semi-open sets by *rσ*, the class of *μ*-regular open sets by *r*, and the class of *μ*-regular closed sets by $r_{c}$. We recall the following facts for their importance in the material of our article.

Proposition 2.1 *Let* (X,μ) *be a space. Then*

*(i)* *[2]* μ⊂α=σ∩π *;*

*(ii)* *[10]* σ∪π⊂b⊂β *.*

Proposition 2.2 ***[10]*** *Let A be a subset of a space* (X,μ) *. Then*

*(i) If A is μ-β-open, then* $c_{\mu }\left(A\right)$ *is μ-regular closed;*

*(ii) If A is both μ-semi-closed and μ-β-open, then A is μ-semi-open.*

Proposition 2.3 *Let* (X,μ) *be a space. Then*

*(i)* *[2]* *Each of the families σ, π, α, and β is a GT;*

*(ii)* *[10]* *The family b is a GT.*

Concerning Proposition 2.3, it is easy to see that each of the families *σ*, *b*, and *β* is a strong GT, while *π* and *α* need not. The next proposition tells, however, that the statements: *μ* is a strong GT, *α* is a strong GT, and *π* is a strong GT are equivalent.

Proposition 2.4 *Let* (X,μ) *be a space. Then the following are equivalent:*

*(i)* (X,μ) *is a strong GTS;*

*(ii)* (X,α) *is a strong GTS;*

*(iii)* (X,π) *is a strong GTS.*

Proof (i)⇒(ii) Since (X,μ) is a strong GTS, X∈μ, but μ⊂α, so X∈α. Thus by Proposition 2.3(i), (X,α) is a strong GTS.

(ii)⇒(iii) Since (X,α) is a strong GTS, X∈α, but *α* ⊂*π*, so X∈π. Thus by Proposition 2.3(i), (X,π) is a strong GTS.

(iii)⇒(i) Since (X,π) is a strong GTS, X∈π, that is $X\subset i_{\mu }\left(c_{\mu }\left(X\right)\right)=i_{\mu }\left(X\right)$. Thus, $X\subset i_{\mu }\left(X\right)$, but $i_{\mu }\left(X\right)\subset X$, so $i_{\mu }\left(X\right)=X$, that is X∈μ. Hence, (X,μ) is a strong GTS. □

At the end of this section, we review some decompositions of *μ*-regular open sets and regular *μ*-semi-open sets.

Proposition 2.5 ***[11]*** *Let A be a subset of a space* (X,μ) *. Then the following are equivalent:*

*(i) A is μ-regular open;*

*(ii) A is μ-open and μ-semi-closed;*

*(iii) A is μ-open and μ-β-closed;*

*(iv) A is μ-α-open and μ-semi-closed;*

*(v) A is μ-α-open and μ-β-closed;*

*(vi) A is μ-preopen and μ-semi-closed.*

Proposition 2.6 ***[10]*** *Let A be a subset of a space* (X,μ) *. Then the following are equivalent:*

*(i) A is μ-regular open;*

*(ii) A is μ-open and μ-b-closed;*

*(iii) A is μ-α-open and μ-b-closed.*

Proposition 2.7 ***[10]*** *Let A be a subset of a space* (X,μ) *. Then the following are equivalent:*

*(i) A is regular μ-semi-open;*

*(ii) A is μ-semi-open and μ-semi-closed;*

*(iii) A is μ-b-open and μ-semi-closed;*

*(iv) A is μ-β-open and μ-semi-closed;*

*(v) A is μ-semi-open and μ-b-closed;*

*(vi) A is μ-semi-open and μ-β-closed.*

## 3 $\bar{\mu }$-open sets

This section is mainly devoted to introducing and studying $\bar{\mu }$-open sets in GTSs.

Definition 3.1 A subset *A* of a space (X,μ) is called $\bar{\mu }$-open if there exists a *μ*-*b*-open set *U* such that $U\subset A\subset c_{\mu }\left(U\right)$; *A* is called $\bar{\mu }$-closed if X﹨A is $\bar{\mu }$-open. The collection of all $\bar{\mu }$-open subsets of a space (X,μ) will be denoted by $\bar{\mu }$.

We study now the relationship between $\bar{\mu }$-open sets and *μ*-*β*-open sets.

Proposition 3.2 *Let A be a* $\bar{\mu }$ *-open subset of a space* (X,μ) *. Then A is μ-β-open.*

Proof Since *A* is $\bar{\mu }$-open, there exists a *μ*-*b*-open set *U* such that $U\subset A\subset c_{\mu }\left(U\right)$. Thus, $c_{\mu }\left(U\right)=c_{\mu }\left(A\right)$, but *U* is *μ*-*β*-open as b⊂β, so it follows by Proposition 2.2(i) that $c_{\mu }\left(U\right)$ is *μ*-regular closed, and therefore,

$$A\subset c_{\mu }\left(U\right)=c_{\mu }\left(i_{\mu }\left(c_{\mu }\left(U\right)\right)\right)=c_{\mu }\left(i_{\mu }\left(c_{\mu }\left(A\right)\right)\right)\text{.}$$

Hence, *A* is *μ*-*β*-open. □

Corollary 3.3 *Let A be a subset of a space* (X,μ) *. If A is* $\bar{\mu }$ *-open, then* $c_{\mu }\left(A\right)$ *is μ-regular closed.*

Proof Follows from Propositions 2.2(i) and 3.2. □

Corollary 3.4 *Let A be a subset of a space* (X,μ) *. If A is both μ-semi-closed and* $\bar{\mu }$ *-open, then A is μ-semi-open.*

Proof Follows from Propositions 2.2(ii) and 3.2. □

The following two examples show that the converse of Proposition 3.2 need not be true in general.

Example 3.5 Let U={{1,2,3},{3,4,5},{5,6,7}}, and let μ={⋃V:V⊂U} and X={1,2,3,4,5,6,7,8}. Then *μ* is a GT on *X* and (X,μ) is not a strong GTS. If A={1,2,4}, then it is easy to see that *A* is *μ*-*β*-open but not $\bar{\mu }$*-*open.

Example 3.6 Let U={{1,2,3},{3,4,5},{5,6,7}}, and let μ={⋃V:V⊂U} and X={1,2,3,4,5,6,7}. Then (X,μ) is a strong GTS. If A={1,2,4}, then it is easy to see that *A* is *μ*-*β*-open but not $\bar{\mu }$*-*open.

Recall that a subset *A* of a topological space (X,τ) is called preopen [12] (resp. *β*-open [13], *b*-open [14]) if $A\subset \mathrm{Int}\;\bar{A}$ (resp. $A\subset \bar{\mathrm{Int}\;\bar{A}}$, $A\subset \mathrm{Int}\;\bar{A}\cup \bar{\mathrm{Int}\;A}$), where $\bar{A}$ and Int *A* stand respectively for the closure of *A* in *X* and the interior of *A* in *X*.

Next, we study the relationship between *μ*-*b*-open sets and $\bar{\mu }$-open sets. To proceed, we recall the following lemma; we will, however, prove it for the convenience of the reader.

Lemma 3.7 **[15]** Let (X,τ) be a topological space and *A* be a subset of *X*. Then *A* is *β*-open if and only if there exists a preopen set *P* such that $P\subset A\subset \bar{P}$.

Proof **Necessity.** Suppose that *A* is *β*-open, that is $A\subset \bar{\mathrm{Int}\;\bar{A}}$. Then $A=\bar{\mathrm{Int}\;\bar{A}}\cap \left(A\cup \left(X﹨\bar{A}\right)\right)$. Let $U=\mathrm{Int}\;\bar{A}$ and $D=A\cup \left(X﹨\bar{A}\right)$. Then clearly *U* is open and *D* dense. Now,

$$U\cap D\subset A\subset \bar{U}=\bar{U\cap D}\text{.}$$

Let P=U∩D. Then $P\subset A\subset \bar{P}$. Also, since *U* is open and *D* is dense, it follows by Proposition 2.1 of [16] that *P* is preopen.

**Sufficiency.** Assume that there exists a preopen set *P* such that $P\subset A\subset \bar{P}$. Then clearly, $\bar{A}=\bar{P}$. Since *P* is preopen, $P\subset \mathrm{Int}\;\bar{P}$, and therefore,

$$A\subset \bar{A}=\bar{P}\subset \bar{\mathrm{Int}\;\bar{P}}=\bar{\mathrm{Int}\;\bar{A}}\text{.}$$

Thus, $A\subset \bar{\mathrm{Int}\;\bar{A}}$, that is *A* is *β*-open. □

Proposition 3.8 *Let* (X,μ) *be a topological space and A be a subset of X. Then A is* $\bar{\mu }$ *-open if and only if A is μ-β-open.*

Proof The necessity follows from Proposition 3.2. Assume that *A* is *μ*-*β*-open. Then by Lemma 3.7, there exists a *μ*-preopen set *P* such that $P\subset A\subset c_{\mu }\left(P\right)$. Since *P* is *μ*-preopen, *P* is *μ*-*b*-open. Thus, *A* is $\bar{\mu }$-open. □

Remark 3.9 Clearly, every *μ*-*b*-open set is $\bar{\mu }$-open; however, the converse need not be true, in general, even for topological spaces as in a topological space a $\bar{\mu }$-open set means *μ*-*β*-open (Proposition 3.8), and it is known that in a topological space, a *β*-open set need not be *b*-open in general (see [14]).

Now, we are ready to characterize spaces (X,μ) in which β=b by means of $\bar{\mu }$-open sets.

Corollary 3.10 *Let* (X,μ) *be a space. Then* β=b *if and only if* $\bar{\mu }=\beta$ *and* $b=\bar{\mu }$ *.*

Proof By Proposition 3.2 and Remark 3.9, $b\subset \bar{\mu }\subset \beta$. Thus, the equivalence becomes clear. □

The following proposition tells that the collection of $\bar{\mu }$-open sets forms a strong GT.

Proposition 3.11 *Let* (X,μ) *be a space. Then* $\bar{\mu }$ *is a strong GT.*

Proof Since ∅ is *μ*-*b*-open and every *μ*-*b*-open set is $\bar{\mu }$-open, ∅ is $\bar{\mu }$-open. Assume that $U_{\alpha }$ is $\bar{\mu }$-open, where α∈Λ. Then for each α∈Λ, there exists a *μ*-*b*-open set $V_{\alpha }$ such that

$$V_{\alpha }\subset U_{\alpha }\subset c_{\mu }\left(V_{\alpha }\right)\text{.}$$

Thus,

$${⋃}_{\alpha \in \Lambda }V_{\alpha }\subset {⋃}_{\alpha \in \Lambda }U_{\alpha }\subset {⋃}_{\alpha \in \Lambda }c_{\mu }\left(V_{\alpha }\right)\subset c_{\mu }\left({⋃}_{\alpha \in \Lambda }V_{\alpha }\right)\text{.}$$

Since $V_{\alpha }$ is *μ*-*b*-open, it follows by Proposition 2.3(ii) that ${⋃}_{\alpha \in \Lambda }V_{\alpha }$ is *μ*-*b*-open. Hence, ${⋃}_{\alpha \in \Lambda }U_{\alpha }$ is $\bar{\mu }$-open. This shows that $\bar{\mu }$ is a GT on *X*. As $X\in b\subset \bar{\mu }$, $\bar{\mu }$ is a strong GT. □

At the end of this section, we study several characterizations of $\bar{\mu }$-open sets as well as $\bar{\mu }$-closed sets.

Proposition 3.12 *Let* (X,μ) *be a space and A be a subset of X. Then A is* $\bar{\mu }$ *-closed if and only if there exists a μ-b-closed set F such that* $i_{\mu }\left(F\right)\subset A\subset F$ *.*

Proof $$A\text{ is }\bar{\mu }\text{-closed}\Leftrightarrow X﹨A\text{ is }\bar{\mu }\text{-open}\Leftrightarrow U\subset X﹨A\subset c_{\mu }\left(U\right)\text{, for some }\mu \text{-}b\text{-open set }U\Leftrightarrow X﹨c_{\mu }\left(U\right)\subset A\subset X﹨U\Leftrightarrow i_{\mu }\left(X﹨U\right)\subset A\subset X﹨U\text{.}$$

Letting F=X﹨U, we observe that *F* is *μ*-*b*-closed as *U* is *μ*-*b*-open, and the result follows. □

Proposition 3.13 *Let* (X,μ) *be a space and A be a subset of X. Then A is* $\bar{\mu }$ *-open if and only if* $A\subset c_{\mu }\left(i_{b_{\mu }}\left(A\right)\right)$ *.*

Proof **Necessity.** Since *A* is $\bar{\mu }$-open, there exists a *μ*-*b*-open set *U* such that $U\subset A\subset c_{\mu }\left(U\right)$. Since *U* is *μ*-*b*-open, $U=i_{b_{\mu }}\left(U\right)$. Thus,

$$A\subset c_{\mu }\left(U\right)=c_{\mu }\left(i_{b_{\mu }}\left(U\right)\right)\subset c_{\mu }\left(i_{b_{\mu }}\left(A\right)\right)\text{.}$$

**Sufficiency.** Since $A\subset c_{\mu }\left(i_{b_{\mu }}\left(A\right)\right)$, $i_{b_{\mu }}\left(A\right)\subset A\subset c_{\mu }\left(i_{b_{\mu }}\left(A\right)\right)$. Let $U=i_{b_{\mu }}\left(A\right)$. Then *U* is *μ*-*b*-open, and thus, *A* is $\bar{\mu }$-open. □

Corollary 3.14 *Let* (X,μ) *be a space and A be a subset of X. Then A is* $\bar{\mu }$ *-open if and only if* $c_{\mu }\left(A\right)=c_{\mu }\left(i_{b_{\mu }}\left(A\right)\right)$ *.*

Proof **Necessity.** Since *A* is $\bar{\mu }$-open, it follows from Proposition 3.13 that $A\subset c_{\mu }\left(i_{b_{\mu }}\left(A\right)\right)$. Thus,

$$c_{\mu }\left(A\right)\subset c_{\mu }\left(i_{b_{\mu }}\left(A\right)\right)\subset c_{\mu }\left(A\right)\text{.}$$

Hence, $c_{\mu }\left(A\right)=c_{\mu }\left(i_{b_{\mu }}\left(A\right)\right)$.

**Sufficiency.** Since $c_{\mu }\left(A\right)=c_{\mu }\left(i_{b_{\mu }}\left(A\right)\right)$ and $A\subset c_{\mu }\left(A\right)$, $A\subset c_{\mu }\left(i_{b_{\mu }}\left(A\right)\right)$. Thus by Proposition 3.13, *A* is $\bar{\mu }$-open. □

Corollary 3.15 *Let* (X,μ) *be a space and A be a subset of X. Then*

*(i)* $c_{\mu }\left(i_{b_{\mu }}\left(A\right)\right)$ *is* $\bar{\mu }$ *-open;*

*(ii)* $i_{\mu }\left(c_{b_{\mu }}\left(A\right)\right)$ *is* $\bar{\mu }$ *-closed;*

*(iii)* $c_{\mu }\left(i_{b_{\mu }}\left(A\right)\right)\subset A$ *if and only if* $c_{\mu }\left(i_{b_{\mu }}\left(A\right)\right)\subset i_{\bar{\mu }}\left(A\right)$ *;*

*(iv)* $A\subset i_{\mu }\left(c_{b_{\mu }}\left(A\right)\right)$ *if and only if* $c_{\bar{\mu }}\left(A\right)\subset i_{\mu }\left(c_{b_{\mu }}\left(A\right)\right)$ *.*

Proof (i)

$$c_{\mu }\left(i_{b_{\mu }}\left(A\right)\right)=c_{\mu }\left(i_{b_{\mu }}\left(i_{b_{\mu }}\left(A\right)\right)\right)\text{ (as }i_{b_{\mu }}\left(A\right)\text{ is }\mu \text{-}b\text{-open)}\subset c_{\mu }\left(i_{b_{\mu }}\left(c_{\mu }\left(i_{b_{\mu }}\left(A\right)\right)\right)\right)\text{.}$$

Hence by Proposition 3.13, $c_{\mu }\left(i_{b_{\mu }}\left(A\right)\right)$ is $\bar{\mu }$-open.

(ii) Follows from (i) since

$$X﹨i_{\mu }\left(c_{b_{\mu }}\left(A\right)\right)=c_{\mu }\left(X﹨c_{b_{\mu }}\left(A\right)\right)=c_{\mu }\left(i_{b_{\mu }}\left(X﹨A\right)\right)\text{.}$$

(iii) If $c_{\mu }\left(i_{b_{\mu }}\left(A\right)\right)\subset A$, then by (i), $c_{\mu }\left(i_{b_{\mu }}\left(A\right)\right)\subset i_{\bar{\mu }}\left(A\right)$. The converse is clear as $i_{\bar{\mu }}\left(A\right)\subset A$.

(iv) If $A\subset i_{\mu }\left(c_{b_{\mu }}\left(A\right)\right)$, then by (ii), $c_{\bar{\mu }}\left(A\right)\subset i_{\mu }\left(c_{b_{\mu }}\left(A\right)\right)$. The converse is clear as $A\subset c_{\bar{\mu }}\left(A\right)$. □

Corollary 3.16 *Let* (X,μ) *be a space and A be a subset of X. Then A is* $\bar{\mu }$ *-closed if and only if* $i_{\mu }\left(c_{b_{\mu }}\left(A\right)\right)\subset A$ *.*

Proof $$A\text{ is }\bar{\mu }\text{-closed}\Leftrightarrow X﹨A\text{ is }\bar{\mu }\text{-open}\Leftrightarrow X﹨A\subset c_{\mu }\left(i_{b_{\mu }}\left(X﹨A\right)\right)\text{ (by Proposition 3.13)}\Leftrightarrow X﹨c_{\mu }\left(i_{b_{\mu }}\left(X﹨A\right)\right)\subset A\Leftrightarrow i_{\mu }\left(X﹨i_{b_{\mu }}\left(X﹨A\right)\right)\subset A\Leftrightarrow i_{\mu }\left(c_{b_{\mu }}\left(A\right)\right)\subset A\text{.}$$

 □

Corollary 3.17 *Let* (X,μ) *be a space and A be a subset of X. Then A is* $\bar{\mu }$ *-closed if and only if* $i_{\mu }\left(A\right)=i_{\mu }\left(c_{b_{\mu }}\left(A\right)\right)$ *.*

Proof **Necessity.** Since *A* is $\bar{\mu }$-closed, it follows from Corollary 3.16 that $i_{\mu }\left(c_{b_{\mu }}\left(A\right)\right)\subset A$. Thus,

$$i_{\mu }\left(c_{b_{\mu }}\left(A\right)\right)\subset i_{\mu }\left(A\right)\subset i_{\mu }\left(c_{b_{\mu }}\left(A\right)\right)\text{.}$$

Hence, $i_{\mu }\left(A\right)=i_{\mu }\left(c_{b_{\mu }}\left(A\right)\right)$.

**Sufficiency.** Since $i_{\mu }\left(A\right)=i_{\mu }\left(c_{b_{\mu }}\left(A\right)\right)$ and $i_{\mu }\left(A\right)\subset A$, $i_{\mu }\left(c_{b_{\mu }}\left(A\right)\right)\subset A$. Thus by Corollary 3.16, *A* is $\bar{\mu }$-closed. □

Proposition 3.18 *Let* (X,μ) *be a space and A be a subset of X. Then A is* $\bar{\mu }$ *-open if and only if there exists a* $\bar{\mu }$ *-open set B such that* $B\subset A\subset c_{\mu }\left(B\right)$ *.*

Proof **Necessity.** Take B=A.

**Sufficiency.** Since *B* be $\bar{\mu }$-open, there exists a *μ*-*b*-open set *U* such that $U\subset B\subset c_{\mu }\left(U\right)$. Thus, $c_{\mu }\left(B\right)=c_{\mu }\left(U\right)$, but $B\subset A\subset c_{\mu }\left(B\right)$, so,

$$U\subset B\subset A\subset c_{\mu }\left(U\right)\text{.}$$

Hence, *A* is $\bar{\mu }$-open. □

Corollary 3.19 *Let* (X,μ) *be a space and A be a subset of X. Then A is* $\bar{\mu }$ *-closed if and only if there exists a* $\bar{\mu }$ *-closed set B such that* $i_{\mu }\left(B\right)\subset A\subset B$ *.*

Proof **Necessity.** Take B=A.

**Sufficiency.** Since $i_{\mu }\left(B\right)\subset A\subset B$,

$$X﹨B\subset X﹨A\subset X﹨i_{\mu }\left(B\right)=c_{\mu }\left(X﹨B\right)\text{.}$$

Since X﹨B is $\bar{\mu }$-open, it follows from Proposition 3.18 that X﹨A is $\bar{\mu }$-open, that is *A* is $\bar{\mu }$-closed. □

## 4 Applications

The primary purpose of this section is to obtain some decompositions of *μ*-regular open sets and regular *μ*-semi-open sets using $\bar{\mu }$-open sets. We also provide several characterizations of spaces in which $\bar{\mu }=\pi$.

The following proposition provides decompositions of *μ*-regular open sets using $\bar{\mu }$-open sets.

Proposition 4.1 *Let A be a subset of a space* (X,μ) *. Then the following are equivalent:*

*(i) A is μ-regular open;*

*(ii) A is μ-open and* $\bar{\mu }$ *-closed;*

*(iii) A is μ-α-open and* $\bar{\mu }$ *-closed.*

Proof Follows from Proposition 2.5, Proposition 2.6 as $b\subset \bar{\mu }\subset \beta$. □

Corollary 4.2 *Let A be a subset of a space* (X,μ) *. Then the following are equivalent:*

*(i) A is μ-regular closed;*

*(ii) A is μ-closed and* $\bar{\mu }$ *-open;*

*(iii) A is μ-α-closed and* $\bar{\mu }$ *-open.*

The next proposition provides decompositions of regular *μ*-semi-open sets using $\bar{\mu }$-open sets.

Proposition 4.3 *Let A be a subset of a space* (X,μ) *. Then the following are equivalent:*

*(i) A is regular μ-semi-open;*

*(ii) A is* $\bar{\mu }$ *-open and μ-semi-closed;*

*(iii) A is μ-semi-open and* $\bar{\mu }$ *-closed.*

Proof Follows from Proposition 2.7 as $b\subset \bar{\mu }\subset \beta$. □

We will now give several characterizations of spaces in which $\bar{\mu }=\pi$. To proceed, we recall the following proposition.

Proposition 4.4 ***[10]*** *For a space* (X,μ) *, the following are equivalent:*

*(i)* α=σ *;*

*(ii)* σ⊂π *;*

*(iii)* β=π *;*

*(iv)* b=π *;*

*(v)* rσ⊂π *;*

*(vi)* r=rσ *;*

*(vii)* $r_{c}\subset \pi$ *;*

*(viii)* $r_{c}\subset \mu$ *.*

Corollary 4.5 *For a space* (X,μ) *, the following are equivalent:*

*(i)* α=σ *;*

*(ii)* σ⊂π *;*

*(iii)* β=π *;*

*(iv)* b=π *;*

*(v)* rσ⊂π *;*

*(vi)* r=rσ *;*

*(vii)* $r_{c}\subset \pi$ *;*

*(viii)* $r_{c}\subset \mu$ *;*

*(ix)* $\bar{\mu }=\pi$ *.*

Proof Follows from Proposition 4.4 as $\pi \subset b\subset \bar{\mu }\subset \beta$. Observe that if $\bar{\mu }=\pi$, then b=π, and that if β=π, then $\bar{\mu }=\pi$. Thus,

(ix)⇒(iv)⇔(iii)⇒(ix).

 □

At the end of this section, it might be convenient to raise the following four questions.

Question 1 Characterize spaces (X,μ) in which $\bar{\mu }=\beta$?

Question 2 Characterize spaces (X,μ) in which $\bar{\mu }=b$?

Question 3 Characterize spaces (X,μ) in which β=σ?

Question 4 Characterize spaces (X,μ) in which $\bar{\mu }=\sigma$?

**Conclusion** In a GTS (X,μ), the class $\bar{\mu }$ consisting of all $\bar{\mu }$-open sets is a strong GT; it is strictly placed between the class *b* consisting of all *μ*-*b*-open sets and the class *β* consisting of all *μ*-*β*-open sets. Decompositions, of *μ*-regular open sets as well as regular *μ*-semi-open sets using $\bar{\mu }$-open sets, were possible. Characterizing spaces in which $\bar{\mu }=\pi$, where *π* is the class of all *μ*-preopen sets, was also reachable; meanwhile, characterizing spaces, in which $\bar{\mu }=\beta$ and spaces in which $\bar{\mu }=b$ as well as spaces in which β=σ and spaces in which $\bar{\mu }=\sigma$, would be interesting.

## CRediT authorship contribution statement

Mohammad Shawqi Sarsak: Conceived and designed the experiments; Performed the experiments; Analyzed and interpreted the data; Contributed reagents, materials, analysis tools or data; Wrote the paper.

## Funding statement

This research did not receive any specific grant from funding agencies in the public, commercial, or not-for-profit sectors.

## Declaration of Competing Interest

The authors declare no conflict of interest.

## Data Availability

No data was used for the research described in the article.
